# Use of gelatin puzzle phantoms to teach medical students isolated ultrasound transducer movements and fundamental concepts

**DOI:** 10.1186/s12909-020-1937-8

**Published:** 2020-01-29

**Authors:** Lauren M. Maloney, Peggy A. Seidman, Kristen M. Zach, Neera K. Tewari, Matthew F. Tito, Christopher R. Page

**Affiliations:** 1 Department of Emergency Medicine, Stony Brook University Hospital, HSC Level 4 Room 050, Stony Brook, NY 11794 USA; 2 Department of Anesthesiology and Perioperative Medicine, 11100 Euclid Ave, Cleveland, OH 44106 USA; 3 Department of Emergency Medicine, 600 Gresham Drive, Raleigh Building Room 304, Norfolk, VA 23507 USA; 4 Department of Anesthesiology, Stony Brook University Hospital, HSC Level 4 Room 060, Stony Brook, NY 11794 USA

**Keywords:** Medical student, Ultrasound education, Ultrasound phantom, Medical education, Ultrasound, Gelatin

## Abstract

**Background:**

Psychomotor skills related to the use of medical ultrasound are a fundamental, but often overlooked component of this ubiquitous medical imaging technology. Although discussions of image production/orientation, sonographic planes, and imaging/scanning techniques are common in existing literature, these discussions rarely address practical skills related to these basic concepts. The cognitive load of transducer movements and machine operation, in conjunction with learning the ultrasound representation of anatomy, may overwhelm a novice learner. Our goal was to develop and evaluate a set of ultrasound puzzle phantoms for students to use as they learn isolated, specific transducer movements and sonographic concepts. We intentionally created phantoms that contain objects that are likely familiar to students to reduce the cognitive load associated with simultaneously learning the ultrasound interpretation of anatomy.

**Methods:**

This preliminary evaluation of our novel, homemade, gelatin ultrasound puzzle phantoms was performed using pretests and posttests obtained by scanning an assessment phantom, and student questionnaires. Two phases of training and testing occurred with feedback from Phase 1 allowing for refinement of the puzzles and techniques for testing. Skills taught and evaluated included probe rotation, depth assessment, sliding, and tilting.

**Results:**

Twenty-eight students attended the Phase 1 training session with positive trends in students’ abilities to use rotation, sliding, and tilting to answer questions, while only depth showed statistically significant improvements (*p* = 0.021). Overall students agreed the experience a productive use of time (86%), was beneficial (93%), and would recommend to others (93%). Fifteen (54%) students returned 3 months later. There was no significant decay in skills obtained from the prior training session. In Phase 2, 134 medical students participated, and 76 (57%) completed an online questionnaire. A majority of students agreed they had a better understanding of rotation (83%), depth (80%), sliding (88%) and tilting (55%). Similar to Phase 1, many students (75%) felt the experience was beneficial.

**Conclusions:**

This preliminary study gave us insight into student opinions, as well as information to guide future scalability and development of additional ultrasound puzzle phantoms to aid in medical student education of isolated transducer movements and sonographic concepts prior to imaging human anatomy.

## Background

Most existing literature describing specific medical schools’ curricula begin with introducing students to the physics of ultrasonography and technical use of the machine. Then they progress to practical skills sessions at the bedside or simulation environments to image specific anatomy [1–4]. While some authors note discussion of image production/orientation, sonographic planes, and imaging/scanning techniques [5–11], these discussions do not allow for reproducible detail, nor do such concepts seem to be the isolated focus of a practical skills session. The psychomotor skills involved with obtaining a 2-dimensional ultrasound image and successfully creating a 3-dimensional conceptualization of the structure being scanned requires having both baseline knowledge of the images that are being sought, as well as an understanding of how transducer movement impacts the images produced [12]. As such, the cognitive load of transducer movement and machine operation, in conjunction with image interpretation in the context of recognizing tissue anatomy or pathology, may overwhelm a novice learner [13].

In this study, our goal was to develop and evaluate a set of homemade, gelatin ultrasound puzzle phantoms that contain familiar objects to reduce the cognitive load associated with conceptualizing a new structure (e.g. a body organ). Each ultrasound puzzle phantom isolated a single transducer movement or sonographic concept to allow students to see how each movement impacts the 2-dimensional ultrasound representation of the 3-dimensional puzzle structure. Learning objectives for the puzzle phantoms were: [1] rotate the probe to see the long (longitudinal) and short (transverse) axes of a tubular structure, [2] count the number of “steps” present and determine the depth of the superficial surface of an object resting on one of the steps, [3] understand how sliding the probe shows different 2D cross-sectional images of a 3D structure, and [4] understand how tilting the probe changes the image of the object being scanned. The puzzle phantoms were trialed with a small group of medical students (Phase 1), then scaled up for a full class of medical students (Phase 2). Improvement in student understanding of the concepts was based on score improvements on pretests and posttests that asked students questions about the structure of an unknown object contained within a visually opaque assessment puzzle phantom.

## Methods

We conducted a prospective evaluation of our ultrasound puzzle phantoms using pretests and posttests, obtained by scanning an assessment puzzle phantom, as well as student questionnaires. Data was linked by unique student-created, anonymous identifiers.

This study took place at the Stony Brook University School of Medicine in New York, USA, a suburban allopathic medical school. Medical students in the graduating classes of 2019 (Phase 1) and 2021 (Phase 2) participated.

During Phase 1, four ultrasound machines (Mindray M7, Mindray North America, Mahwah, NJ) were available during each class. Linear 7.5 MHz ultrasound transducers (Mindray 7L4s, Mindray North America, Mahwah, NJ) were used during the practice and assessment scans. During Phase 2, an additional ultrasound machine (SonoSite M Turbo, SonoSite, Inc., Bothell, WA), with linear probe (13-6 MHz, SonoSite, Inc., Bothell, WA) was available.

### Phase 1

Four transparent instructional puzzle phantoms were developed using readily available objects. Cooled transparent gelatin (three envelopes of unflavored gelatin (Knox; Kraft Foods, Northfield, IL) for every 240 mL of hot water) was poured into the containers. Containers were gently agitated to release bubbles, and refrigerated overnight.

**Fig. 1:**
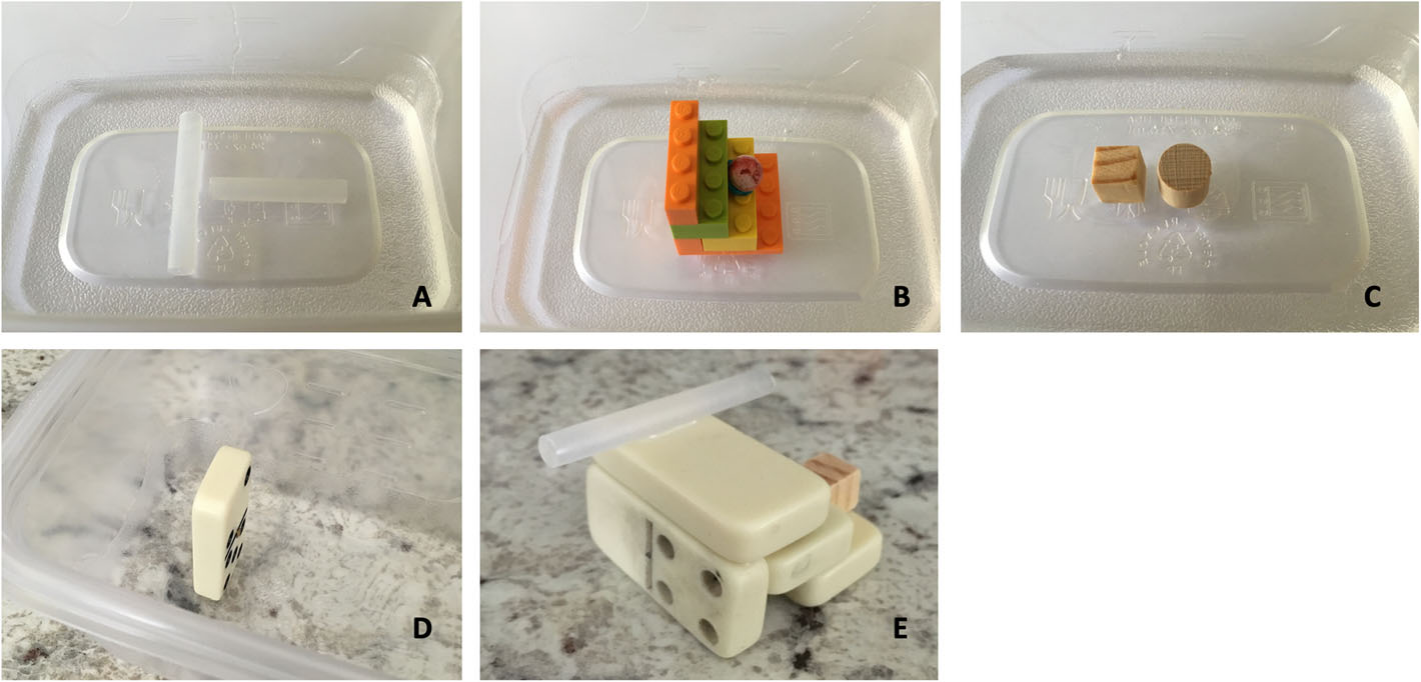
Puzzle phantoms used during Phase 1. **a** Rotation puzzle. **b** Depth puzzle. **c** Sliding puzzle. **d** Tilting puzzle. **e** Assessment phantom

1. Rotation puzzle phantom: spinal needle covers were attached in the shape of a T to the bottom of a plastic container, assuring the distance between the two perpendicular pieces allowed for simultaneous observation with a linear ultrasound probe (Fig. 1a).
2. Depth puzzle phantom: a set of steps [3, 4] were made using LEGOs® (The LEGO Group, Denmark), and a large acrylic spherical bead was attached to one of the steps (Fig. 1b).
3. Sliding puzzle phantom: ¼” diameter square and rounded wooden dowels approximately ¼” long were attached to the bottom of a container assuring that both objects would both be simultaneously observable in the footprint of the linear ultrasound probe (Fig. 1c).
4. Tilting puzzle phantom: a domino was attached perpendicularly to the bottom of a container (Fig. 1d).

The assessment puzzle phantom used for pretest and posttests contained a structure combining all four instructional elements (Fig. 1e) and was filled with visually opaque gelatin [14] (For every 600 mL water: 2 envelopes of unflavored gelatin (Knox; Kraft Foods, Northfield, IL), 2 boxes of black cherry gelatin (Jell-O; Kraft Foods Group, INC, Northfield, IL), and 2 boxes of berry blue gelatin (Jell-O; Kraft Foods Group, INC, Northfield, IL)).

On the day of the training session, students scanned the assessment puzzle phantom and answered questions about the hidden structures (Additional file 1). Student then rotated through four stations, each with an ultrasound machine, an instructional puzzle phantom, and an example of the goal ultrasound image with the station’s learning objective (Additional file 1). Each student spent 5 min at each station. Throughout the experience, students completed a worksheet that encouraged timely reflection on the skill they just performed (Additional file 1), and faculty members rotated between the stations to help optimize student learning. Students then rescanned the assessment puzzle phantom, answered the same questions (Additional file 1), and completed a survey (Additional file 1). Three months later, students were asked to return and rescan the assessment puzzle phantom (Additional file 1).

### Phase 2

**Fig. 2:**
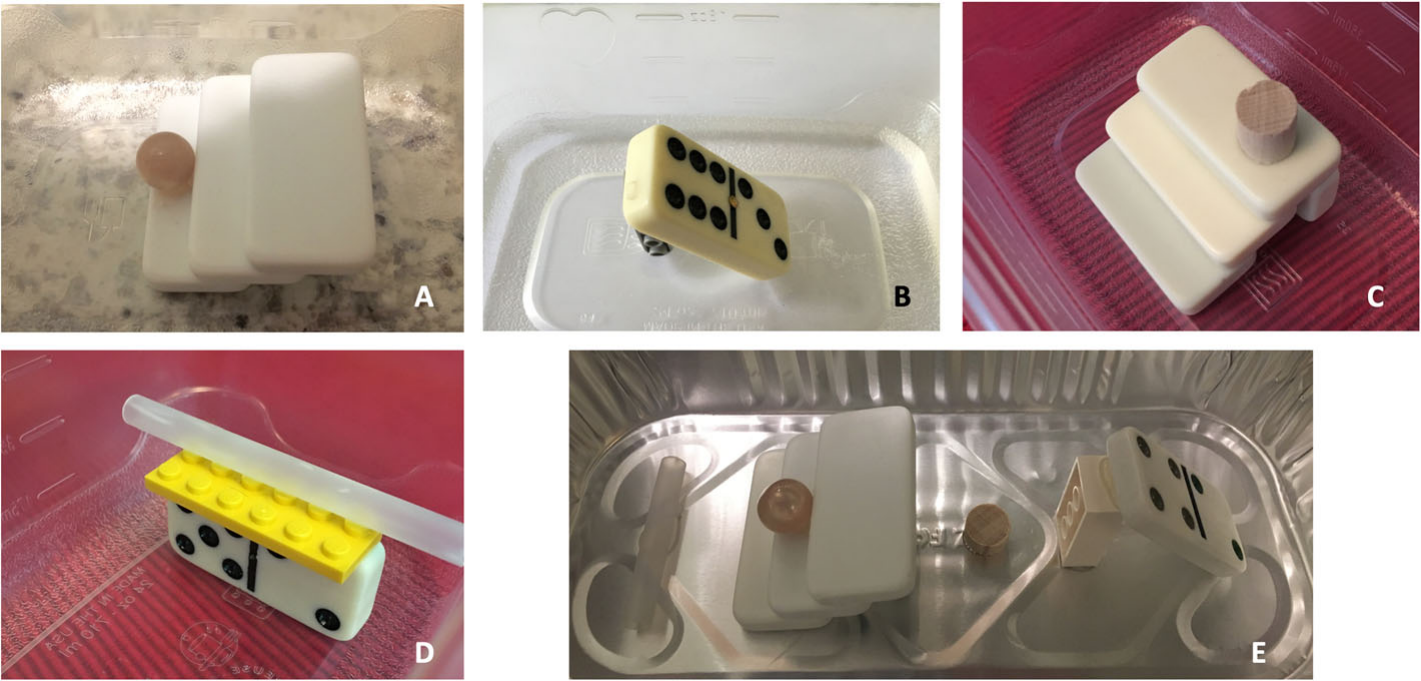
Puzzle phantom modifications used during Phase 2. **a** Revised depth puzzle. **b** Revised tilting puzzle. **c** New puzzle combining sliding and depth. **d** New puzzle combining tilting and rotation. **e** New assessment phantom

Based on feedback from Phase 1, the depth and tilting instructional puzzle phantoms were adjusted (Fig. 2a and b). Two new instructional puzzle phantoms, each integrating two of the previously isolated skills (Fig. 2c shows the combination of sliding and depth; Fig. 2d shows the combination of tilting and rotation), were developed. Additionally, the assessment puzzle phantom was altered (Fig. 2e), and a different recipe that produced a more resilient gelatin was used (for every 240 mL boiling water: 3 envelopes of unflavored gelatin (Knox; Kraft Foods, Northfield, IL) and 10 g of Metamucil Fiber Supplement (Procter & Gamble, Cincinnati, OH)).

During the training session, 2–3 students spent 30 min with a single faculty member at a station that had an ultrasound machine and a set of the puzzle phantoms. Initially students scanned the assessment puzzle phantom and answered questions about the hidden structures (Additional file 1). Students then scanned the instructional puzzle phantoms with an instructor present to offer feedback about their technique, as well as to demonstrate the goal ultrasound image. Additionally, students completed the same worksheet as in Phase 1, to encourage timely reflection on the skills (Additional file 1). At the end of the session, students rescanned the assessment puzzle phantom, answered questions about the structure it contained (Additional file 1), and received an anonymous survey via email (Additional file 1).

### Statistical analysis

In this feasibility study, the sample size for Phase 1 was determined a priori to be 30, based on available ultrasound machines and faculty. Sample size for Phase 2 was dictated by the number of students enrolled in the medical school graduating class of 2021.

Responses about the structure contained within the assessment puzzle phantom were graded binarily. Each question required using rotation, tilting, depth, or sliding to determine the answer. Each question was graded with a “yes” if the student answered it correctly, or “no” otherwise. Student responses to each question were tracked though different test iterations.

Quantitative survey data was analyzed using standard descriptive statistics. For Phase I, pretest data and immediate posttest data were compared using McNemar’s Test. Of the students who returned for the delayed post-test, a related-samples Cochran’s Q test was used to compare their pretest, immediate posttest, and delayed posttest performance. In Phase 2, pretest and posttest data were compared using McNemar’s Test, with *p* < 0.05 for statistical significance. All statistical analysis was performed using SPSS Statistics Faculty Pack 25 (IBM Corporation, Armonk, NY).

## Results

### Phase 1

In total, 30 students enrolled in the course, 28 attended the training session, and 15 (54%) returned for the follow-up assessment puzzle phantom scan 3 months later. Six instructor hours were needed to teach 28 students.

Of the 28 who attended the training session, 25 (89%) indicated prior ultrasound experience, which largely involved spending several minutes scanning an ultrasound phantom looking for embedded fruit the year before. As seen in Table 1, when comparing pretest and immediate posttest responses, based on scanning the assessment puzzle phantom, while there were positive trends in students’ abilities to use rotation, sliding, and tilting to answer questions, only depth showed statistically significant improvements (*p* = .021). Most students agreed that: there was enough time to complete the instructional puzzles (*n* = 26, 93%), the course was a productive use of time (*n* = 24, 86%), they would recommend the experience to others (*n* = 26, 93%), and that the skills learned would benefit them as future medical students (*n* = 26, 93%).

**Table 1:** Students’ ability to correctly answer questions about the structure contained within the assessment puzzle phantom during Phase 1. A total of 28 medical students were assessed

Psychomotor Skill	Pretest n(%)	Immediate Posttest n(%)	*p*
Draw domino face (tilting)	6 (21%)	6 (21%)	1
Determine direction domino facing (tilting)	2 (7%)	6 (21%)	0.125
Identify shape of item on set of steps (sliding)	10 (36%)	12 (43%)	0.804
Determine depth of superficial surface of item on steps (depth)	2 (7%)	10 (36%)	0.021
Determine orientation of tubular structure (rotating)	6 (21%)	13 (46%)	0.092

Of the fifteen (54%) students who returned 3 months later, there was no significant decay in skill when the immediate posttest results were compared to the delayed posttest results (Table 2).

**Table 2:** Students’ ability to correctly answer questions about the structure contained within the assessment puzzle phantom during Phase 1, 3 months after the training course (Delayed Posttest). Fifteen medical students participated in the delayed posttest; their specific performance on the immediate posttest is included (Immediate Posttest)

Psychomotor Skill	Immediate Posttest n(%)	Delayed Posttest n(%)	*p*
Draw domino face (tilting)	3 (20%)	9 (60%)	0.028
Determine direction domino facing (tilting)	5 (33%)	5 (33%)	0.105
Identify shape of item on set of steps (sliding)	7 (47%)	8 (53%)	0.584
Determine depth of superficial surface of item on steps (depth)	7 (47%)	6 (40%)	0.122
Determine orientation of tubular structure (rotating)	6 (40%)	7 (47%)	0.236

### Phase 2

In total, 134 medical students participated in the training sessions, and 76 (57%) completed the online questionnaire. Thirty instructor hours were needed to teach all 134 students.

Based on scanning the assessment puzzle phantom, students demonstrated positive trends in their abilities to use rotation, sliding, and tilting to answer questions about the structure of an unknown object. Improvements in tilting were statistically significant (*p* < 0.001) (Table 3).

**Table 3:** Students’ ability to correctly answer questions about the structure contained within the assessment puzzle phantom during Phase 2. A total of 134 students were assessed

Psychomotor Skill	Pretest n(%)	Immediate Posttest n(%)	*p*
Draw domino face (tilting)	9 (7%)	25 (19%)	<.001
Identify shape of item on set of steps (sliding)	71 (53%)	81 (60%)	0.174
Determine depth of superficial surface of item on steps (depth)	24 (18%)	27 (20%)	0.720
Recognize the axis (transverse, longitudinal) of a tubular structure (rotating)	80 (60%)	83 (63%)	0.788
Determine orientation of tubular structure (rotating)	69 (52%)	78 (58%)	0.328

Of those returning a questionnaire, 7% (*n* = 5) reported prior experience with ultrasonography. A majority of students who completed the survey agreed they felt they had a better understanding of rotation (83%, *n* = 63), depth (80%, *n* = 61), sliding (88%, *n* = 67), and tilting (55%, *n* = 42). Additionally, a majority of students agreed the session was a productive use of time (70%, *n* = 53), and that the skills they learned would benefit them in the future as medical students (75%, *n* = 57).

### Cost

The cost of supplies for each instructional puzzle phantom was approximately $8.00. The cost of supplies for each assessment puzzle phantom was approximately $16.00.

## Discussion

Nicholls et al. [12] eloquently deconstructs the psychomotor skills universal to all ultrasonography into “ [1] being able to view 3-dimensional anatomy in real time on a 2-dimensional screen [2]; moving a transducer in multiple planes [3]; scanning an organ in a minimum of 2 orthogonal planes; [and] [4] depicting the optimal image of a structure for a given clinical scenario.”

We have developed a preliminary set of puzzle phantoms that offer students the opportunity to conceptualize the different ultrasound representations of a 3-dimensional structure without cognitive overload, as well as accompanying teaching materials. We deliberately used common objects in transparent puzzle phantoms to allow students to more closely focus on specific transducer movements and sonographic concepts as they relate to the overall 3-dimensional structure, instead of conflating it with complex anatomy recognition which can lead to cognitive overload [13, 15, 16].

In addition to using transparent puzzle phantoms so that learners can visually connect the structure to its ultrasound representation, the goal ultrasound image was provided with each puzzle. This was done to facilitate the students’ complex motor neuron analysis and resulting mental construct of the object being scanned [12]. Finally, we emphasized using consistent standard terms for each transducer movement to reduce confusion of our novice learners and improve their communication with others in this increasingly important skill.

After the training sessions, students demonstrated improved abilities to use depth and specific transducer movements to answer questions about the structure of an unknown object. These abilities were shown to resist decay after 3 months. As we do not have a specific explanation for the variation in statistically significant improvements in the use of depth in Phase 1 and tilting in Phase 2, that will need to be further investigated in a future study.

As a novel method of teach basic concepts of ultrasound imaging, an iterative approach is necessary to hone the design of the puzzles being used in order to optimize student ability to learn the principles that are being taught. Although the concepts may seem trivial to experienced providers, past and future iterations are important in order to provide clarity to the inexperienced learner. Improvement from Phase 1 to Phase 2 can be attributed to several factors. First, the gelatin mix was altered as described to make the puzzle phantoms more resilient. There was an additional ultrasound machine available for Phase 2 which improved workflow. Finally, as many of the same instructors were involved there was likely some refinement in their ability to explain these concepts to students in the context of this teaching method. Low skill acquisition could conceivably be improved by additional time being allowed for students to practice with the puzzle phantoms both alone or with instructor supervision. By making the puzzle phantoms available to the students outside of normal class time additional improvements in skill acquisition and retention could occur.

Positive student survey responses likely represent, that at this early point in their training, the students appreciated the opportunity to gain exposure in what they realize is an increasing utilized imaging modality across many specialties.

As a result of our collective lessons learned from student feedback and performance, puzzle phantom production, and resource constraints, going forward we recommend a potential hybrid between our Phase 1 and Phase 2 designs. Five stations, each with a dedicated ultrasound machine is a functional number to decrease wait time between actual learning on models, and allow ongoing student engagement with the session. The first two stations would consist of two of the instructional puzzle phantoms (rotation, sliding, depth, tilting/fanning), with both the transparent and opaque versions, accompanied by the goal ultrasound image and station learning objectives. The next two stations would each have one of the more advanced instructional puzzle phantoms that combine two psychomotor skills (Fig. 2c and d), again utilizing transparent and opaque versions, in addition to the goal ultrasound image and station learning objects. The final station would consist of the assessment puzzle phantom. Students would rotate sequentially through each of the 5 stations, allowing 5 min per station. At the assessment station, students would be allowed sufficient time to scan the assessment puzzle phantom and answer questions, as well as a quick debrief on the actual structure it contains. Ideally, there would be one instructor per station, though the first four stations could be handled with a minimum of two instructors, each able to supervise two simultaneous stations. Incorporation of a post-course debriefing which reveals the structure contained within the assessment phantom would likely be well received by students and improve retention. Finally, if data were being collected, a sixth station could be added as to allow for a pretest assessment puzzle phantom scan. We would also recommend asking students to complete any course surveys in person at the end of the session, as response rates were rather poor when surveys were sent electronically.

The most significant limitation of this feasibility study was the use of only one cohort of students for each iteration of the puzzle phantoms. Additionally, time and ultrasound machine availability hindered the use of all the designed puzzles in Phase 2. Furthermore, bias may have been introduced because of the low percentage of students who returned for the delayed assessment scan in Phase 1, and low student survey response rate in Phase 2. Finally, due to the resources allocated to this feasibility study, we were not able to demonstrate if the use of these puzzle phantoms correlated with improved clinical performance of ultrasonography.

## Conclusions

Current medical student ultrasound education largely consists of students learning the physics of ultrasound, and then immediately performing ultrasound exams at a simulated or actual patient bedside [1–4]. We offer our description of the types of homemade gelatin phantoms we made, as well as the accompanying learning materials, as a springboard for other educators to use when considering incorporating a stepping stone into this progression, such that novice students are able to practice isolated transducer movements and fundamental sonographic concepts with a reduced cognitive load.

## Supplementary information

**Additional file 1.** “Electronic Appendixes”: This file contains the accompanying educational material including assessments, station instructions, and course surveys.

## Data Availability

Data are available from the corresponding author on reasonable request.

## Abbreviation

USA: United States of America

## References

[1] Brown B, Adhikari S, Marx J, Lander L, Todd GL (2012). Introduction of ultrasound into gross anatomy curriculum: perceptions of medical students. J Emerg Med..

[2] Hammoudi N, Arangalage D, Boubrit L, Renaud MC, Isnard R, Collet JP (2013). Ultrasound-based teaching of cardiac anatomy and physiology to undergraduate medical students. Arch Cardiovasc Dis.

[3] Amini R, Stolz LA, Gross A, O'Brien K, Panchal AR, Reilly K (2015). Theme-based teaching of point-of-care ultrasound in undergraduate medical education. Intern Emerg Med.

[4] Dinh VA, Frederick J, Bartos R, Shankel TM, Werner L (2015). Effects of ultrasound implementation on physical examination learning and teaching during the first year of medical education. J Emerg Med.

[5] Siegel-Richman Y, Kendall J (2018). Establishing an ultrasound curriculum in undergraduate medical education: how much time does it take?. J Ultrasound Med.

[6] Wong I, Jayatilleke T, Kendall R, Atkinson P (2011). Feasibility of a focused ultrasound training programme for medical undergraduate students. Clin Teach.

[7] Bahner DP, Adkins EJ, Hughes D, Barrie M, Boulger CT, Royall NA (2013). Integrated medical school ultrasound: development of an ultrasound vertical curriculum. Crit Ultrasound J.

[8] Rao S, van Holsbeeck L, Musial JL, Parker A, Bouffard JA, Bridge P (2008). A pilot study of comprehensive ultrasound education at the Wayne State University School of Medicine: a pioneer year review. J Ultrasound Med.

[9] Hoppmann RA, Rao VV, Bell F, Poston MB, Howe DB, Riffle S (2015). The evolution of an integrated ultrasound curriculum (iUSC) for medical students: 9-year experience. Crit Ultrasound J.

[10] Heinzow HS, Friederichs H, Lenz P, Schmedt A, Becker JC, Hengst K (2013). Teaching ultrasound in a curricular course according to certified EFSUMB standards during undergraduate medical education: a prospective study. BMC Med Educ.

[11] Hoppmann RA, Rao VV, Poston MB, Howe DB, Hunt PS, Fowler SD (2011). An integrated ultrasound curriculum (iUSC) for medical students: 4-year experience. Crit Ultrasound J.

[12] Nicholls D, Sweet L, Hyett J (2014). Psychomotor skills in medical ultrasound imaging: an analysis of the core skill set. J Ultrasound Med.

[13] Jamniczky HA, McLaughlin K, Kaminska ME, Raman M, Somayaji R, Wright B (2015). Cognitive load imposed by knobology may adversely affect learners' perception of utility in using ultrasonography to learn physical examination skills, but not anatomy. Anat Sci Educ.

[14] Maloney L, Zach K, Page C, Tewari N, Tito M, Seidman P (2017). Integration of a low-cost introductory ultrasound curriculum into existing procedural skills education for preclinical medical students. J Ultrasound Med.

[15] Yoo MC, Villegas L, Jones DB (2004). Basic ultrasound curriculum for medical students: validation of content and phantom. J Laparoendosc Adv Surg Tech A.

[16] Tanious SF, Cline J, Cavin J, Davidson N, Coleman JK, Goodmurphy CW (2015). Shooting with sound: optimizing an affordable ballistic gelatin recipe in a graded ultrasound phantom education program. J Ultrasound Med.

