# Supplementary material for: Use of gelatin puzzle phantoms to teach medical students isolated ultrasound transducer movements and fundamental concepts
Source: BMC Med Educ. 2020 Jan 29;20:26. doi: 10.1186/s12909-020-1937-8 (PMC6988293; doi:10.1186/s12909-020-1937-8)
Supplement: Supplementary file 1 — Additional file 1. “Electronic Appendixes”: This file contains the accompanying educational material including assessments, station instructions, and course surveys. [file 12909_2020_1937_MOESM1_ESM.docx]

**Electronic Appendix A**

**Phase 1 Assessment Phantom - Student Guide**

Identification #: ________________________ ☐ Pre-Course

☐ Immediate Post-Course

☐ Delayed Post-Course

- You have two minutes to scan an unknown puzzle.
- There is a set of steps; on two steps there are objects.
- At the top of the steps, there is a tubular structure. On a deeper step there is an object whose shape needs to be identified.
- The back side of the steps are supported by a domino whose divotted face is visible.

1. With the indicator at 12 o’clock, draw the face of the domino that is perpendicular to the surface of the phantom:


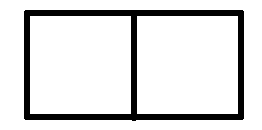


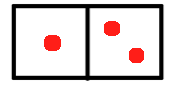
Example:

1. Which direction are the divots on the surface oriented towards?
   - - 12 o’clock
     - 3 o’clock
     - 6 o’clock
     - 9 o’clock
2. The object on the deeper step is a:
   - - sphere
     - cube
     - cylinder

The depth of the top surface of this object is: ____ cm

1. In order to see the transverse (short) axis of the tubular structure, the probe indicator is at:
   - - 12 o’clock
     - 9 o’clock
2. Please check the statement that best applies:
   - - I needed additional time to complete this
     - I had just the right amount of time to complete this
     - I finished with more than 30 seconds to spare

**Electronic Appendix B**

**Instructions for Phase 1 Stations**

**
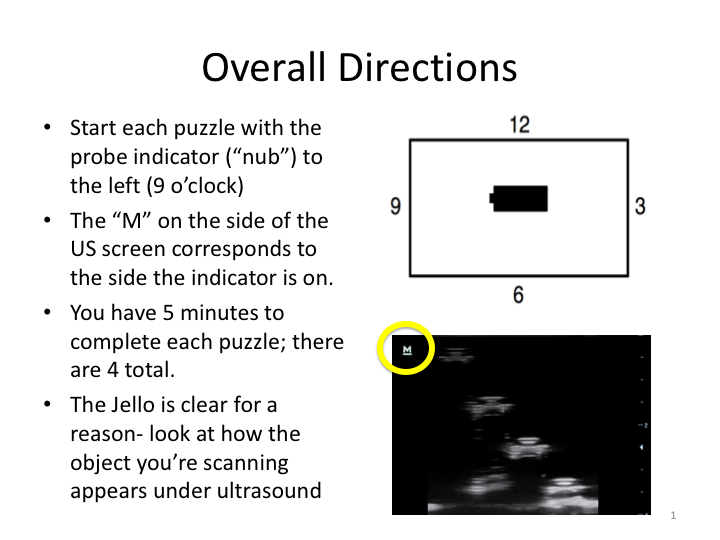
**

**
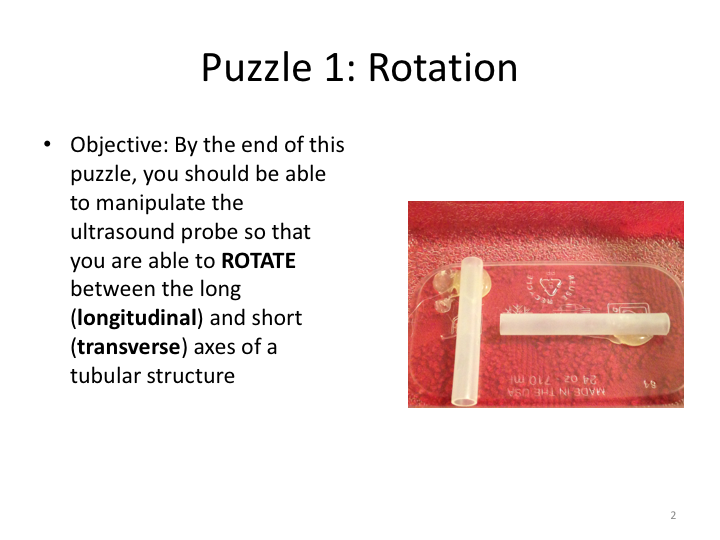
**

**
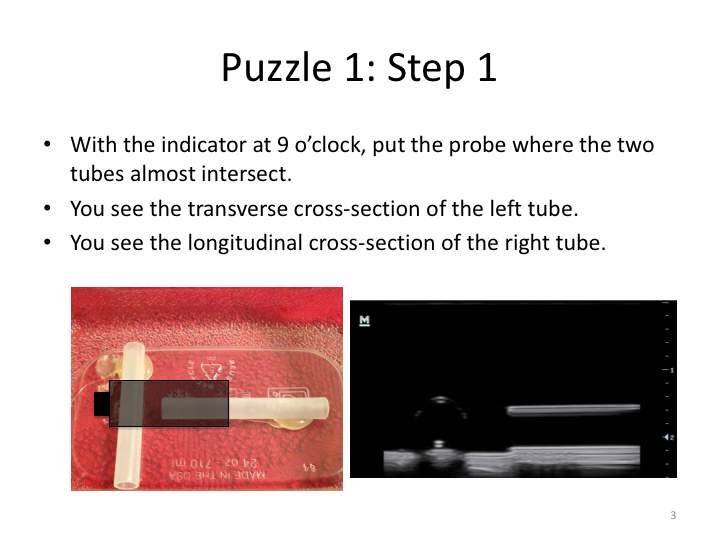
**

**
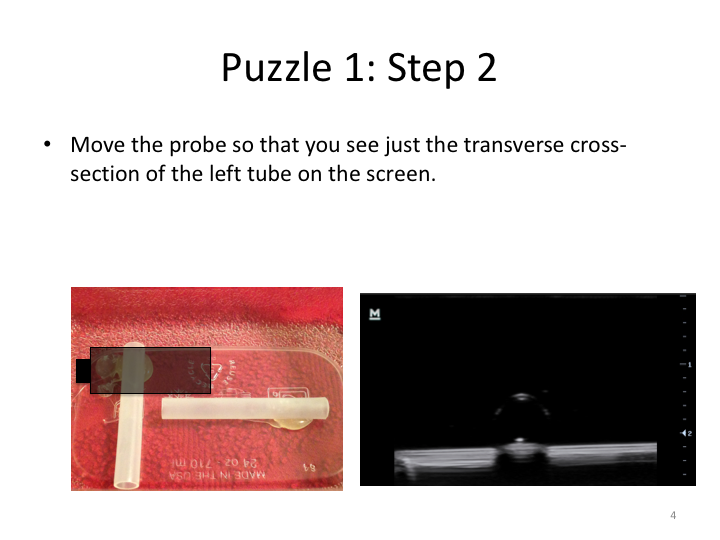
**

**
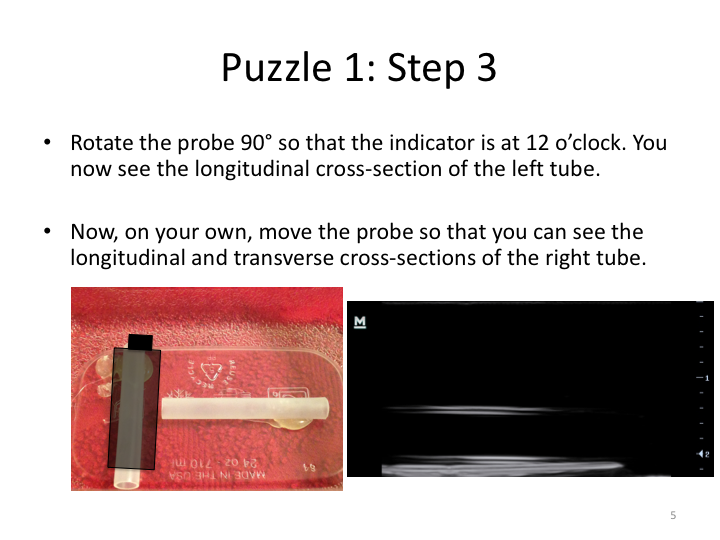
**

**
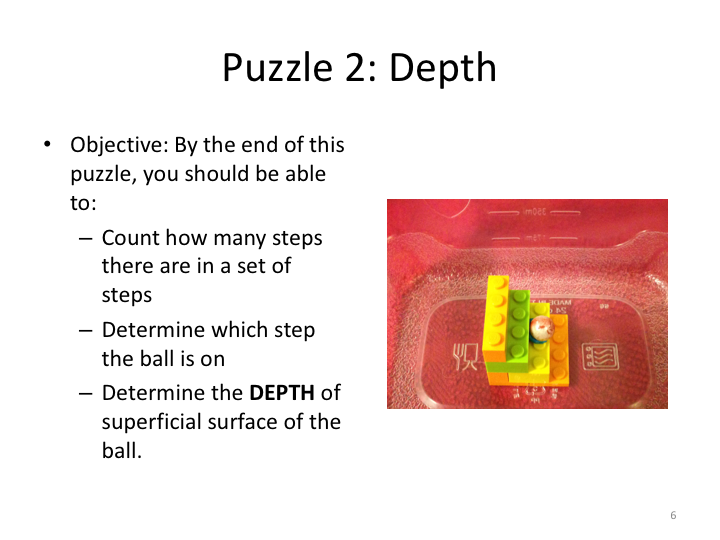
**

**
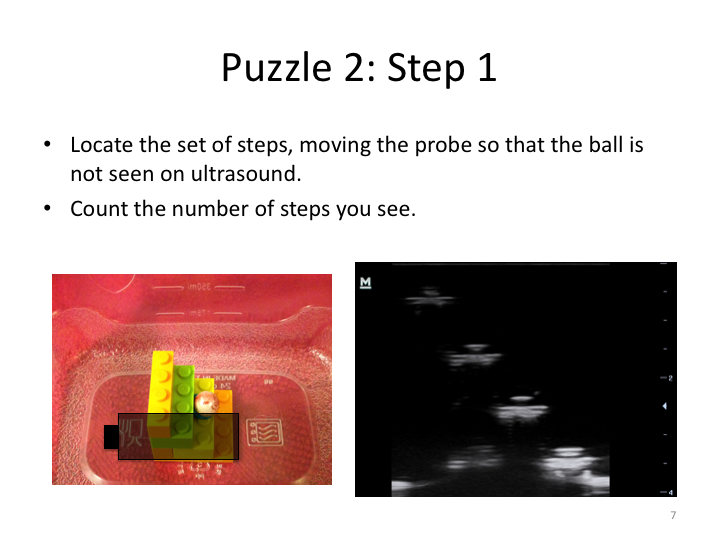
**

**
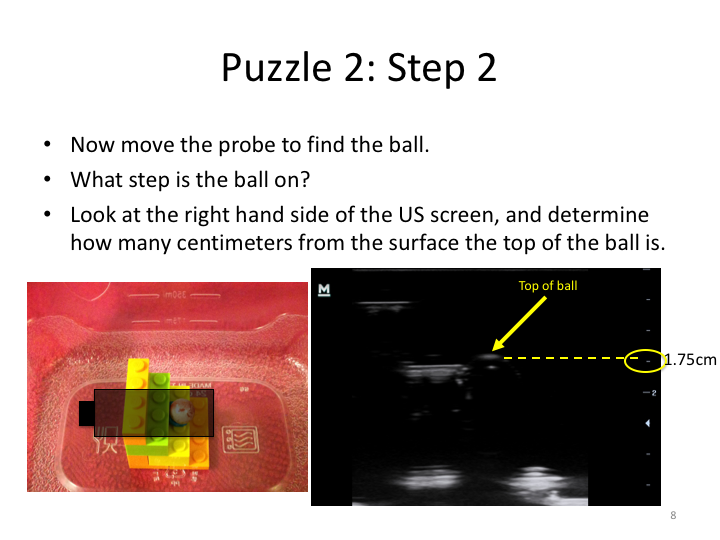
**

**
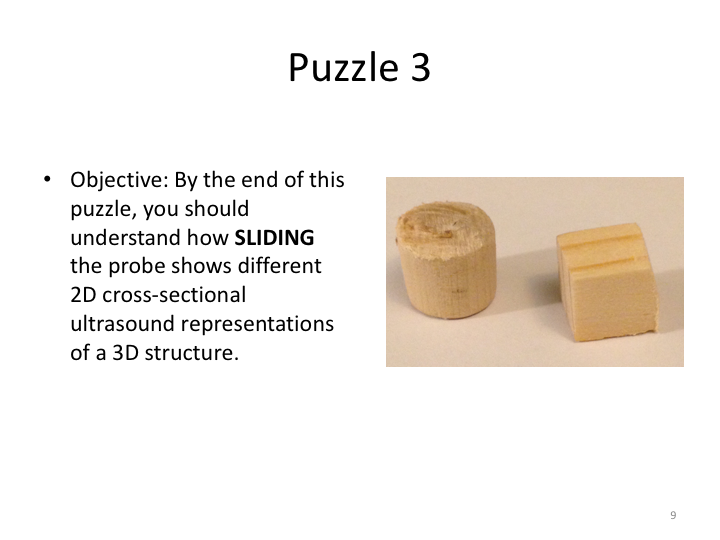
**

**
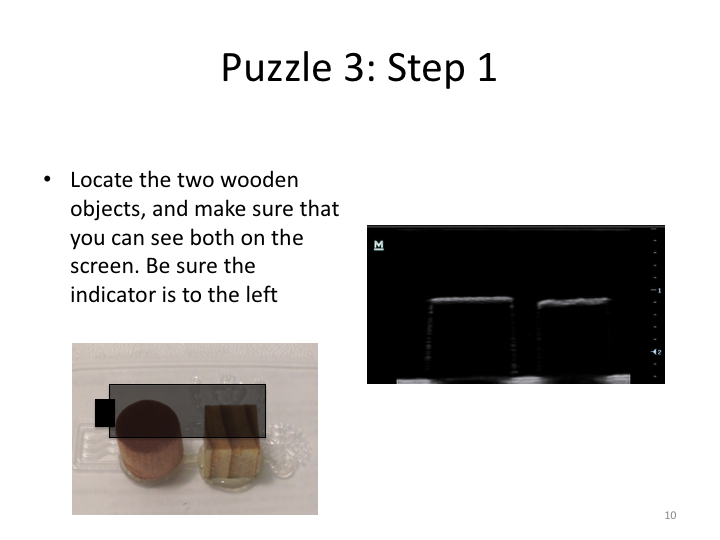
**

**
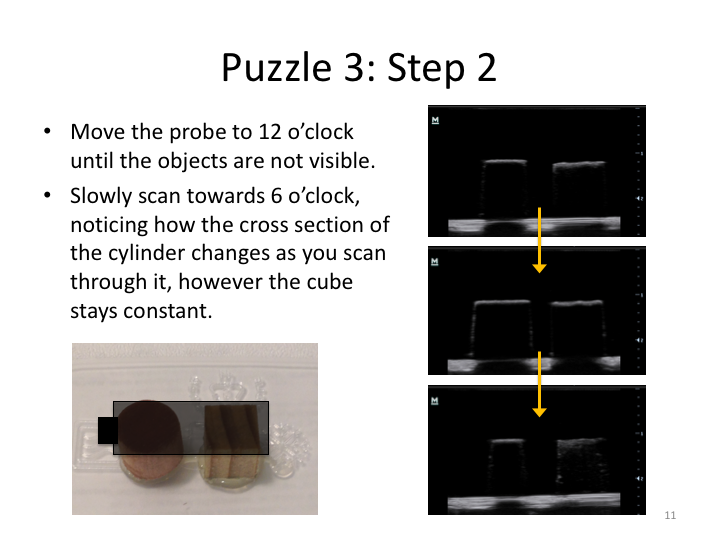
**

**
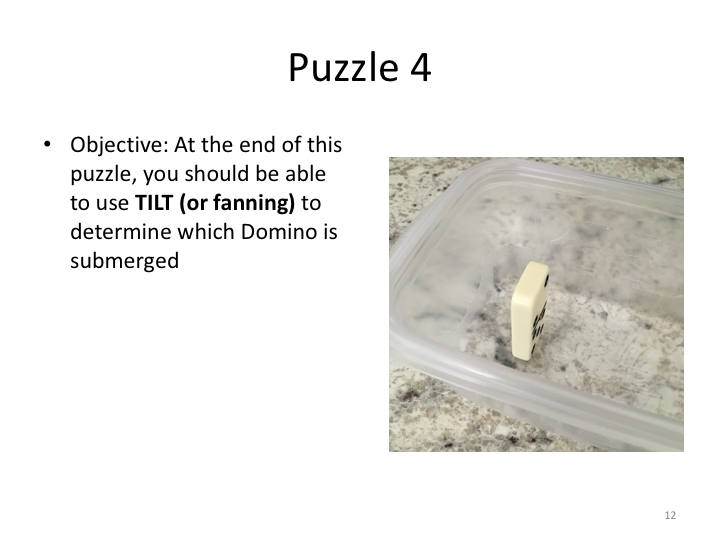
**

**
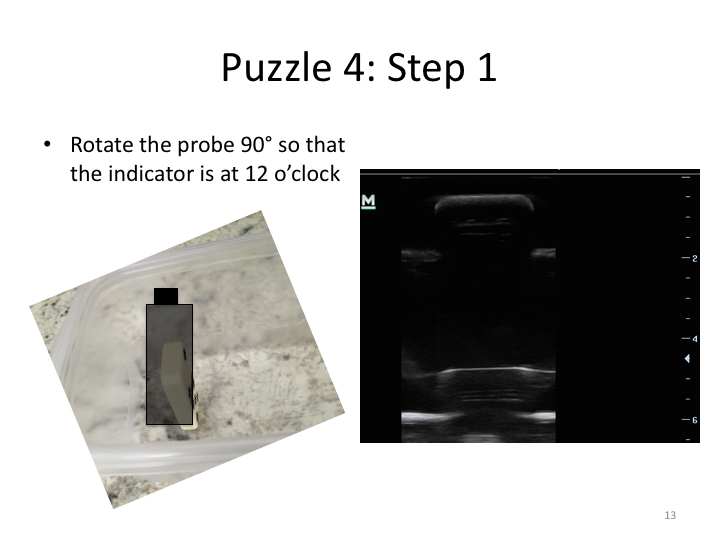
**

**
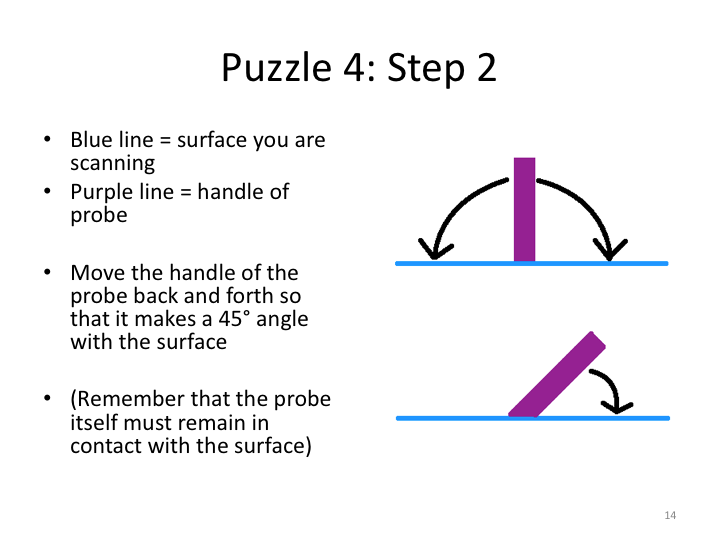
**

**
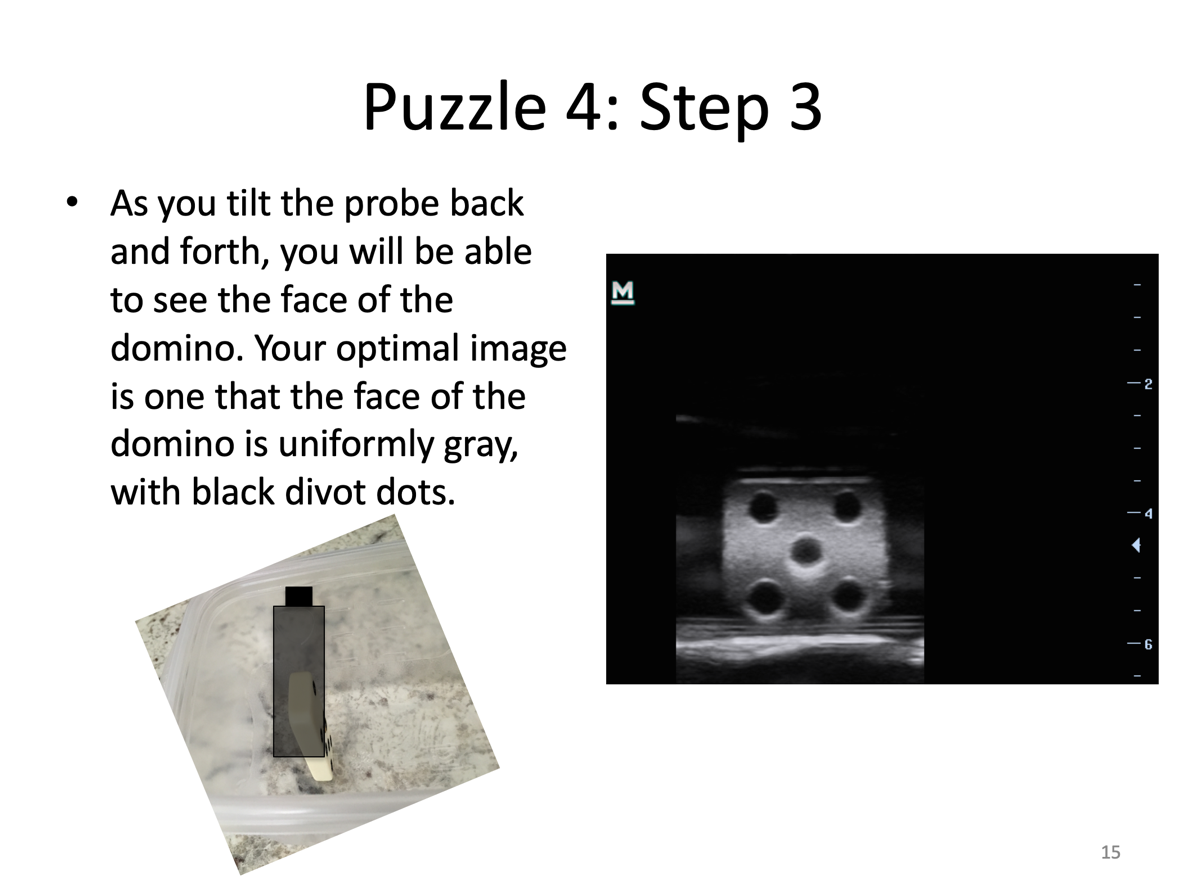
**

**Electronic Appendix C**

**Student Course Worksheet Identification #: __________**

**Puzzle 1:**

Draw the transverse (short axis) Draw the longitudinal (long axis)

representation of a tubular structure: representation of a tubular structure:

**Puzzle 2:**


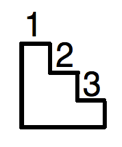


- Using the example to the right, please draw out and number the steps.
- Draw in the ball on the appropriate step.
- The depth of the most superficial surface of the ball is approximately ____________ cm.

**Puzzle 3:**

Please draw what you see on the US screen (the 2D cross section), when the probe is aligned with the center of a cylinder and a cube.

CYLINDER CUBE

**Puzzle 4:**

- Please draw what you see on the face of the domino. Please note that the deeper part of the domino should correspond to the left side of the drawing.

DEEP SUPERFICIAL

- Which direction are the divots on the surface of the domino facing?
  - - 12 o’clock
    - 3 o’clock
    - 6 o’clock
    - 9 o’clock

**Electronic Appendix D**

**Phase 1 Course Survey**

|  | Strongly  Disagree |  |  | Strongly  Agree |
| --- | --- | --- | --- | --- |
| I had enough time to complete each puzzle during the course. | 1 | 2 | 3 | 4 |
| I had enough time to complete the puzzle during the post-course evaluation | 1 | 2 | 3 | 4 |
| I feel as though this was a productive use of my time | 1 | 2 | 3 | 4 |
| The goals and objectives of the course were clear | 1 | 2 | 3 | 4 |
| The goals and objectives of the course were achieved | 1 | 2 | 3 | 4 |
| I would recommend this experience to others | 1 | 2 | 3 | 4 |
| I think the skills I learned will benefit me in my career as a medical student | 1 | 2 | 3 | 4 |

Have you used an ultrasound machine before? If yes, please explain how.

If this course were to be run again in the future, what improvements would you suggest?

Is there anything that was particularly helpful/positive about this course?

**Electronic Appendix E**

**Phase 2 Pre-Course Assessment Puzzle - Student Guide**

What is your DAY of Birth? __ __

What is the first two letters of your mother’s maiden name? __ __

What is the first two letters of your high school mascot? __ __

- You have one minute to scan a challenge puzzle.
- Scan from left to right, with the indicator pointing towards 12 o’clock, to find four objects.

Object 1:

1. What axis of the tube do you see?
   - Longitudinal Axis
   - Transverse Axis
2. If the indicator was pointing towards 9 o’clock, what axis of the tube would you see?
   - Longitudinal Axis
   - Transverse Axis

Object 2:

1. What is the depth of most superficial surface of the spherical object that is on the steps?

_________ cm

Object 3:

1. What is the shape of this structure?
   - Cylinder
   - Cube

Object 4:

1. Draw the face of the domino:


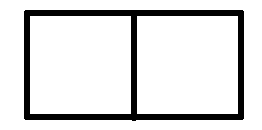


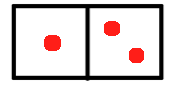
Example:

1. Please check the statement that best applies:
   - - I needed additional time to complete this
     - I had just the right amount of time to complete this
     - I finished with more than 30 seconds to spare

**Electronic Appendix F**

**Phase 2 Post-Course Assessment Puzzle- Student Guide**

What is your DAY of Birth? __ __

What is the first two letters of your mother’s maiden name? __ __

What is the first two letters of your high school mascot? __ __

- You have one minute to scan a challenge puzzle.
- Scan from left to right, with the indicator pointing towards 12 o’clock, to find four objects.

Object 1:

1. What axis of the tube do you see?
   - Longitudinal Axis
   - Transverse Axis
2. If the indicator was pointing towards 9 o’clock, what axis of the tube would you see?
   - Longitudinal Axis
   - Transverse Axis

Object 2:

1. What is the depth of most superficial surface of the spherical object that is on the steps?

_________ cm

Object 3:

1. What is the shape of this structure?
   - Cylinder
   - Cube

Object 4:

1. Draw the face of the domino:


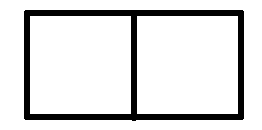


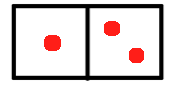
Example:

1. Please check the statement that best applies:
   - - I needed additional time to complete this
     - I had just the right amount of time to complete this
     - I finished with more than 30 seconds to spare

**Electronic Appendix G**

**Phase 2 Course Survey**

|  | Strongly  Disagree |  |  |  | Strongly  Agree |
| --- | --- | --- | --- | --- | --- |
| I had enough time to complete each puzzle during the class | 1 | 2 | 3 | 4 | 5 |
| I have a better understanding of how rotating the probe changes the image I see | 1 | 2 | 3 | 4 | 5 |
| I have a better understanding of how to determine how deep a structure is using ultrasound | 1 | 2 | 3 | 4 | 5 |
| I have a better understanding of how tilting or fanning the probe changes the image I see | 1 | 2 | 3 | 4 | 5 |
| I have a better understanding of how sliding the probe changes the images I see | 1 | 2 | 3 | 4 | 5 |
| I feel as though this was a productive use of my time | 1 | 2 | 3 | 4 | 5 |
| The goals and objectives of the class were clear | 1 | 2 | 3 | 4 | 5 |
| The goals and objectives of the class were achieved | 1 | 2 | 3 | 4 | 5 |
| I would recommend this experience to others | 1 | 2 | 3 | 4 | 5 |
| I think the skills I learned will benefit me in my career as a medical student | 1 | 2 | 3 | 4 | 5 |

When this class runs again in the future, what improvements would you suggest?

Is there anything that was particularly helpful/positive about this class?
